# Transformation of Breast Reconstruction via Additive Biomanufacturing

**DOI:** 10.1038/srep28030

**Published:** 2016-06-15

**Authors:** Mohit P. Chhaya, Elizabeth R. Balmayor, Dietmar W. Hutmacher, Jan-Thorsten Schantz

**Affiliations:** 1 Institute of Health and Biomedical Innovation, Queensland University of Technology, Brisbane, Australia; 2 Department of Plastic and Hand Surgery, Klinikum Rechts der Isar, Technische Universität München, Munich, Germany; 3 Institute for Advanced Study, Technische Universität München, Munich, Germany; 4 School of Chemical and Biomedical Engineering, Nanyang Technological University, Singapore

## Abstract

Adipose tissue engineering offers a promising alternative to current breast reconstruction options. However, the conventional approach of using a scaffold in combination with adipose-derived precursor cells poses several problems in terms of scalability and hence clinical feasibility. Following the body-as-a-bioreactor approach, this study proposes a unique concept of delayed fat injection into an additive biomanufactured and custom-made scaffold. Three study groups were evaluated: Empty scaffold, Scaffold containing 4 cm^3^ lipoaspirate and Empty scaffold +2-week prevascularisation period. In group 3, of prevascularisation, 4 cm^3^ of lipoaspirate was injected into scaffolds after 2 weeks. Using a well-characterised additive biomanufacturing technology platform, patient-specific scaffolds made of medical-grade-polycaprolactone were designed and fabricated. Scaffolds were implanted in subglandular pockets in immunocompetent minipigs (n = 4) for 24-weeks. Angiogenesis and adipose tissue regeneration were observed in all constructs. Histological evaluation showed that the prevascularisation + lipoaspirate group had the highest relative area of adipose tissue (47.32% ± 4.12) which was significantly higher than both lipoaspirate-only (39.67% ± 2.04) and empty control group (8.31% ± 8.94) and similar to native breast tissue (44.97% ± 14.12). This large preclinical animal study provides proof-of-principle that the clinically applicable prevascularisation and delayed fat-injection techniques can be used for regeneration of large volumes of adipose tissue.

Breast cancer is a major cause of illness for women, with an estimated number of 300,000 new cases diagnosed in 2013^1^,^2^. Unfortunately, currently available clinical treatments and filler materials for breast reconstruction or augmentation sometimes yield unsatisfying results^3^,^4^. Reconstructions using silicone-based implants may lead to complications such as fibrous capsule formation around the implant. In some cases this may be associated with pain, soft tissue irritation via capsular contracture and lead, from a cosmetic point of view, to an undesirable appearance to the breast^5^. Additional corrective surgeries may be required to remove the capsule–resulting in amplified costs for the patient and the healthcare system. A second reconstruction method termed lipotransfer, first described by the German surgeon Neuber^6^, is also used routinely for both reconstructive and aesthetic surgeries. In this case, the surgeon isolates fat from a donor site within the patient’s body via liposuction and injects it back into the breast region. However, without a structural support, the newly injected fat quickly gets remodelled by the body and a significant originally regenerated tissue volume is lost after 2–3 months–thus requiring 3–4 additional lipotransfer sessions before the tissue stabilises^7^. Furthermore, lipotransfer of large amounts of adipose tissue bears the risk of adipose tissue necrosis owing to insufficient vascularisation–ultimately leading to formation of oil cysts. Therefore, impetus has been gradually growing towards regenerative medicine-based approaches for breast reconstruction^8^,^9^,^10^,^11^.

Therapy concepts based on application of adult stem cells were thought to be promising for complete regeneration of breast tissue in the early days in the field; however, those concepts also have considerable disadvantages impeding their clinical translation–ranging from complexities and cost with scaling up of tissue culture to requiring complex GMP-certified laboratories for the processing^12^,^13^,^14^,^15^,^16^,^17^,^18^. Furthermore, it is challenging to efficiently vascularise large clinically relevant breast scaffolds (>75 cc) using cell culture-based concepts.

In order to solve the problems of vascularisation and adipose tissue remodelling in the field of breast tissue engineering, we have devised a unique concept based on the combination of an additive biomanufactured and patient-specific biodegradable scaffold in combination with a delayed fat injection. In this method of implantation, a scaffold additive biomanufactured from medical grade polycaprolactone (mPCL) is first implanted into the implantation site. The well-designed fully interconnected large pore network in combination with a surface etching process^19^ allows the formation of a blood clot inside the scaffold architecture^20^. The clot consists of platelets embedded in a mesh of cross-linked fibrin fibres, together with a growth-factor rich cocktail of fibronectin, vitronectin and thrombospondin. It is well known in the literature that the fibrin network and the associated growth-factor cocktail stimulates a strong angiogenic response and induce highly organised connective tissue to penetrate into the affected region^21^,^22^. After 14 days of implantation, when this angiogenic response is at its peak, fat is isolated from a donor site within the patient’s body and injected into the scaffold (see Fig. 1 for a visualisation of this concept).

From a clinical perspective, the amount of fat that can be harvested from the patient without encountering donor site morbidity depends on the body composition of the patient–whereby a larger volume of fat can be extracted from patients with higher body fat percentage. In this study, based on the expertise of our surgical team and an extensive literature search^23^,^24^,^25^,^26^, 4 cm^3^ of adipose tissue was considered to be the maximum amount that can be harvested from a patient with a very low body fat percentage without encountering donor-site complications. Therefore, a study design was chosen in which the scaffolds were injected with 4 cm^3^ of autologous harvested fat–representing 5.2% of the total volume of the implanted scaffold. We further hypothesise that the presence of a pre-formed bed of connective tissue and vasculature would allow the injected fat to remodel within the highly porous scaffold architecture with minimal tissue necrosis and graft resorption.

This study characterised adipose tissue retention in 75 cm^3^ sized patient-specific mPCL scaffolds subjected to a delayed fat injection implanted in a large animal model (pigs) for a period of 24 weeks. The results from this study move the field towards addressing the important clinical question of how to regenerate clinically relevant volumes of adipose tissue by integrating the clinically routinely applied technique of lipotransfer with scaffold-guided regeneration.

## Results

### Clinical Observations

The surgery and implant placement were tolerated well by all animals and no apparent clinical signs of infection were observed throughout the implantation period except one scaffold was observed to have seroma accumulation in the surgically-created pocket twelve weeks after the initiation of the study. This specimen was therefore excluded from further analysis.

### Scaffold characterisation

**Figure 2:**
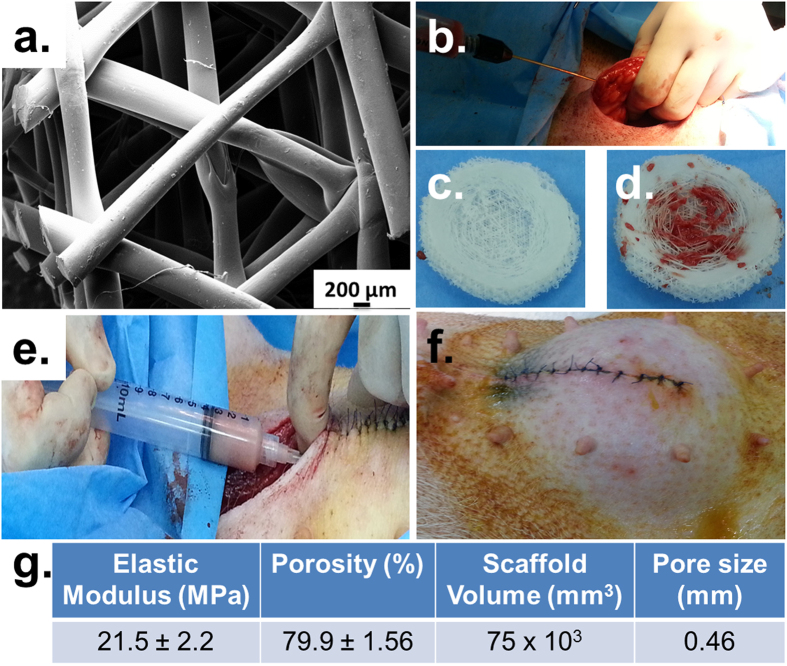
(**a**) Scanning Electron Micrograph of the scaffold showing the struts, pores and pore-interconnections. (**b–f**) Implantation process of the scaffolds. (**b**) Liposuction procedure near the abdominal midline incision. (**c,d**) Process of injecting fat into the pores of the scaffold placed in the lipoaspirate only group. (**c**) shows an empty scaffold while (**d**) shows a completely filled scaffold. (**e**) shows the process of injecting fat into the prevascularisation + lipoaspirate group scaffolds. The scaffolds are placed empty into the implantation site and 2 weeks later, fat is injected into the scaffold pores while the scaffold remains implanted. (**f**) the final form of the scaffolds conforms highly to the natural breast shape (**g**) Physical and mechanical properties of the scaffolds.

The overall geometry of the scaffold was similar to that of a silicone implant used for breast augmentation (Fig. 2). Yet, we have shown the proof of principle to design and manufacture of patient-specific scaffolds (See Supplementary Fig. 1).

### Scaffold explantation and degradation

3 study groups were evaluated in this study:

1. Empty scaffold.
2. Scaffold containing 4 cm^3^ lipoaspirate.
3. Empty scaffold +2 week prevascularisation period. After 2 weeks of prevascularisation, 4 cm^3^ of lipoaspirate was injected into scaffolds.

**Figure 3:**
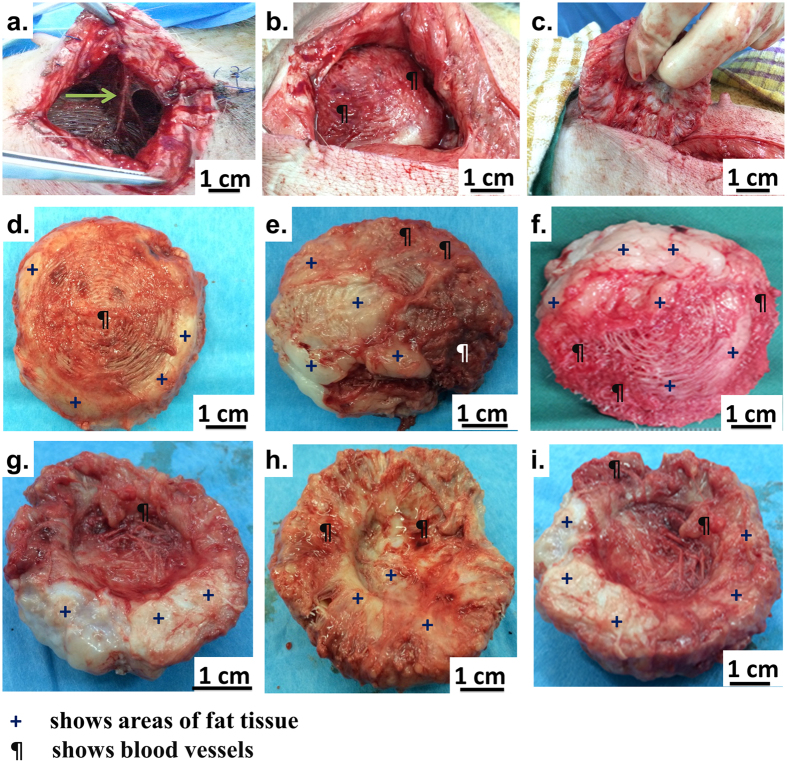
Explantation images showing the integration of TECs with the host tissue. Green arrow point out major blood vessels supplying blood to the TEC. (**d**,**g**) show empty scaffold-only group (**e,h**) show lipoaspirate-only group (**f,i**) show prevascularisation + lipoaspirate group. All scaffolds show good integration with the host tissues and large areas of fat (marked with +) and vascularisation (marked with ^¶^) were observed qualitatively on all scaffolds.

After 6 months of implantation, the Tissue Engineered Constructs (TECs) were retrieved for histological analysis. The scaffolds were well integrated with the surrounding tissue and there was a widespread invasion of host vasculature into the constructs (Fig. 3c). Visual examination revealed that the overall shape of the scaffolds did not change over the 6 month implantation period. All scaffolds showed good integration with the host tissues and large areas of fat and vascularisation were observed qualitatively for all scaffold groups. From a qualitative point of view, prevascularisation + lipoaspirate group (Fig. 3 f,i) had the highest degree of vascularisation and fat tissue deposits, followed by lipoaspirate-only group (Fig. 3e,h). Scaffold-only group showed significant less amounts of adipose tissue (Fig. 3d,g)

### Formation of vascularised adipose tissue

**Figure 4:**
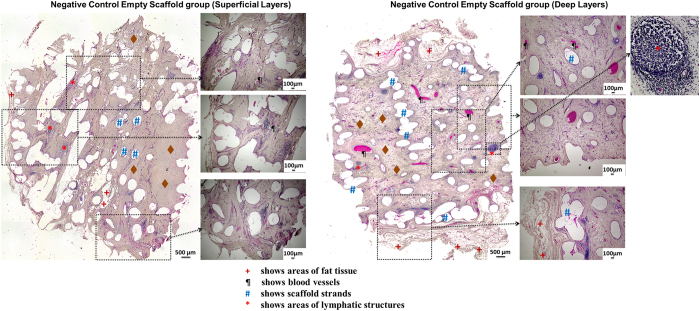
(LEFT) Representative images showing H&E staining of tissue explanted from the empty scaffold group (superficial layers). A majority of the tissue can be identified as being connective tissue and collagen with only very small patches of fat tissue. (RIGHT) Representative images showing H&E staining of tissue explanted from the empty scaffold group (deep layers). Adipose tissue is only seen at the edges of the construct and not in the central regions of the scaffold. Lymphatic structures (right panel, marked by red arrows) were also observed in all groups mainly localised near scaffold strands.

**Figure 5:**
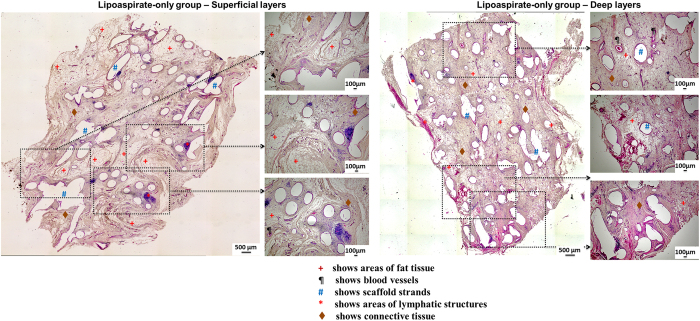
(LEFT) H&E stained sections of lipoaspirate-only group (superficial layers). Overall, a higher percentage of fat tissue compared to overall tissue area, compared to empty scaffold group, was observed in this group. (RIGHT) H&E stained sections of lipoaspirate-only group (deep layers). Deeper layers of the scaffold showed lower relative adipose tissue areas and lower degrees of vascularisation.

**Figure 6:**
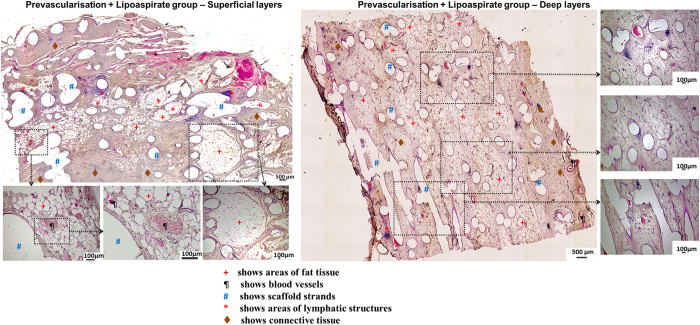
(LEFT) H&E stained sections of prevascularisation + lipoaspirate group (superficial layers). This group showed the highest accumulation of adipose tissue interspersed between connective tissue. Tissue morphology also showed similarities with native tissue. (RIGHT) H&E stained sections of prevascularisation + lipoaspirate group (deep layers). Adipose tissue area was the highest among all other groups. Adipose tissue regions seemed to be better connected to each other and formed interconnected structures.

Figures 4, 5, 6 show representative H&E stained images of all scaffold groups after 24 weeks of implantation. All specimens showed typical ring-like morphology of fat tissue. Overall, multiple areas of well vascularised adipose tissue were found in all scaffold groups.

H&E staining of tissue explanted from the empty scaffold group showed that although the newly infiltrated tissue was highly vascular, a majority of the tissue was connective tissue and collagen with only very small patches of fat tissue (Fig. 4) identified in the micrographs by their typical ring-like morphology and the empty vacuole in the middle of the cell. The deeper layers of the empty scaffolds also showed similar results.

Figure 5 shows the H&E stained sections of lipoaspirate-only group. Overall, a higher percentage of fat tissue compared to overall tissue area (referred from here on as relative tissue area) was observed in this group. The superficial layers of the scaffold especially showed widespread distribution of adipose tissue whose relative tissue area matched closely to that of native breast tissue. However, the deeper layers of the scaffold showed lower relative adipose tissue areas and lower degrees of vascularisation.

The prevascularisation + lipoaspirate group (Fig. 6) showed the highest amount of fat tissue compared to all other groups. There were large highly vascularised regions of fat tissue interspersed between connective tissue. Furthermore, the relative adipose tissue area was also considerably higher in the deeper layers of this group compared to all other groups. These adipose tissue regions seemed to be better connected to each other and formed interconnected structures.

**Figure 7:**
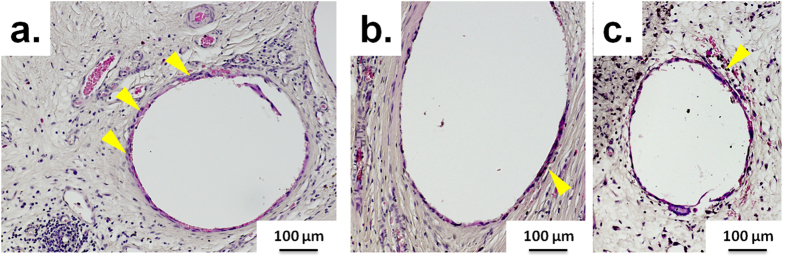
Representative H&E-stained micrographs of regions around the scaffold strands showing non-specific minor granulomatose reactions. (**a**) shows empty scaffold-only group, (**b**) shows lipoaspirate-only group (**c**) shows prevascularisation + lipoaspirate group. Yellow arrows point to macrophages.

While no major signs of chronic inflammation were observed in the tissue sections or in the gross morphology of the constructs, non-specific localised low-grade granulomatose reactions were observed in the vicinity of the localised scaffold strands (Fig. 7). Lymphatic structures (Fig. 4 right panel) and leucocytes were also observed in all groups localised mainly near scaffold strands.

**Figure 8:**
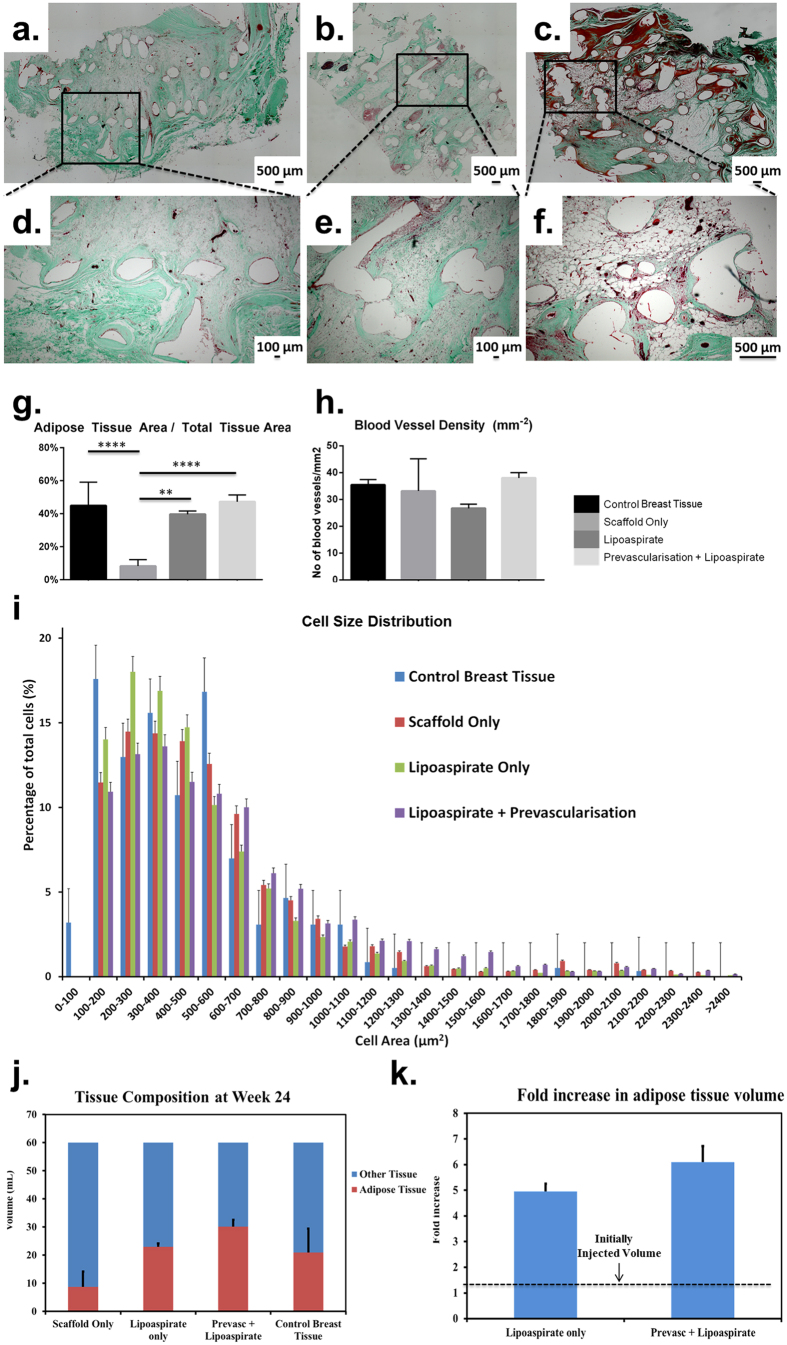
Representative images of Masson’s Trichrome stained tissue sections. Green indicates collagen fibres, red indicates muscle-fibres and dark brown shows cell nuclei. (**a,d**) show empty scaffold group (**b,e**) show prevascularisation + lipoaspirate group (**c,f**) shows lipoaspirate-only group. Besides the adipose tissue, a majority of the tissue filling the pores of the implant consisted of connective and smooth muscle tissue. The smooth muscle layers had the highest thickness in case of the prevascularisation + lipoaspirate group. (**g**) Graph showing the adipose tissue area relative to total tissue area (AA/TA) over 24 weeks. Negative control scaffold-only group had the lowest AA/TA (8.31% ± 8.94) which was significantly lower than lipoaspirate-only (39.67% ± 2.04), prevascularisation + lipoaspirate group (47.32% ± 4.12) and native breast tissue (44.97% ± 14.12) (p < 0.05, p < 0.01 and p < 0.01 respectively). No significant difference in AA/TA was observed between the other groups. (**h**) Graph showing blood vessel density in the tissue sections. Highest blood vessel density was observed in the prevascularisation + lipoaspirate group (38.01/mm^2^ ± 2.02). However the density was not significantly higher than the scaffold-only (33.13/mm^2^ ± 12.03), lipoaspirate-only (26.67/mm^2^ ± 1.6) or control breast tissue (35.45/mm^2^ ± 1.93). (**i**) Histogram showing the distribution of adipose cells by cell surface-area. All histograms were skewed to the right suggesting that majority of cell surface areas lay in 100–700 μm^2^ range. The empty scaffold and lipoaspirate-only groups had a low number of adipose cells with surface areas larger than 800 μm^2^. The prevascularisation + lipoaspirate group showed a more equalised distribution with a significantly large number of cells having a surface area larger than 1000 μm^2^. (**j**) Graph showing tissue composition at week 24. TECs from empty scaffold group contained an estimated 4.99 cm^3^ ( ± 2.71) of adipose tissue, TECs from lipoaspirate-only group contained an estimated 23.85 cm^3^( ± 1.22) of adipose tissue, whereas TECs from prevascularisation + lipoaspirate group contained an estimated 28.391 cm^3^ ( ± 2.48) of adipose tissue. (**k**) Graph showing estimated fold increase in adipose volume compared to initial injected lipoaspirate volume (4 cm^3^). Prevascularisation + Lipoaspirate group had a higher fold increase in adipose volume (6.1 ± 0.62) compared to lipoaspirate-only group (4.95 ± 0.31); however, the difference was not statistically significant (p = 0.143).

To identify the nature and composition of the connective tissue, Masson’s trichrome staining was performed (Fig. 8a–f). Green colour indicates collagen fibres, red colour indicates muscle fibres and dark brown shows cell nuclei. As can be seen from the micrographs, besides the adipose tissue, a majority of the tissue filling the pores of the implant consisted of collagen fibres.

Thin layers of smooth muscle tissue were also observed, however it was only lining the boundaries of the scaffold struts. These smooth muscle layers had the highest thickness in case of the prevascularisation + lipoaspirate group (Fig. 8c).

In order to quantify adipose tissue regeneration, the total area of the adipose tissue relative to the total tissue area was counted on all slides (Fig. 8g). The empty scaffold group (control group) had the lowest relative area of adipose tissue (8.31% ± 8.94) which was significantly lower than both lipoaspirate-only (39.67% ± 2.04) and prevascularisation + lipoaspirate group (47.32% ± 4.12) and also compared to native breast tissue (44.97% ± 14.12) (p < 0.05, p < 0.01 and p < 0.01 respectively). However, there was no statistically significant difference in relative adipose tissue area between the native breast tissue, lipoaspirate-only and prevascularisation + lipoaspirate group.

To quantify neovascularisation, blood vessels were counted on all slides (Fig. 8 h). These blood vessels were identified by a ring/tubular structure, with only those lined with red blood cells included in the count as functional blood vessels. In general, all constructs, including the empty scaffold-only group, showed a substantial ingression of neovascularisation. The highest blood vessel density was observed in the prevascularisation + lipoaspirate group (38.01/mm^2^ ± 2.02), however the density was not statistically significantly higher than the scaffold-only (33.13/mm^2^ ± 12.03), lipoaspirate-only (26.67/mm^2^ ± 1.6) or control breast tissue (35.45/mm^2^ ± 1.93). H&E sections of constructs also showed blood vessel on and parallel to the surface of the constructs, suggesting that new capillaries are likely to have sprouted from these larger vessels that penetrated into the scaffolds.

Quantification of adipose cell area allowed the visualisation of the distribution of different-sized cells as a histogram (Fig. 8i). In all groups, the histograms were skewed to the right suggesting that a majority of adipose cell surface areas lay in the range of 100–700 μm^2^. The distribution of the cell sizes in control breast tissue was considerably different compared to rest of the groups–with the highest percentage of cells in the 100–200, 300–400 and 500–600 μm^2^ range. The empty scaffold and lipoaspirate-only groups had a low number of adipose cells having a surface area larger than 800 μm^2^; whereas, the prevascularisation + lipoaspirate group showed a considerably higher number of cells having a surface area larger than 800 μm^2^.

From data showing the percentage of adipose tissue area relative to total tissue area, the fold increase in adipose tissue volume was calculated (Fig. 8j,k). Prevascularisation + Lipoaspirate group had a higher fold increase in adipose tissue volume (6.1 ± 0.62) compared to lipoaspirate-only group (4.95 ± 0.31); however, the difference was not statistically significant (p = 0.143). Data for empty scaffold group has not been included because lipoaspirate was not injected into these scaffolds.

## Discussion

The ability to generate *de novo* adipose tissue for post-mastectomy breast reconstruction is widely thought to transform the field of breast reconstructive surgery^7^,^8^,^27^,^28^,^29^. Previous long-term rodent studies performed in our laboratory have shown sustained regeneration of high volumes of fat using cell-seeded anatomically shaped scaffolds^30^. These cell-based approaches have merit from a tissue engineering perspective; however, from a clinically driven regenerative medicine point of view they also have disadvantages with problems ranging from scaling up of tissue culture protocols and associated costs and regulatory hurdles. To circumvent these problems while scaling up the volumes of adipose tissue being regenerated, here we have followed the philosophy of implanting an additive biomanufactured scaffold and using the patient’s body as a bioreactor. However, in the absence of a strong adipogenic stimulus, the scaffold gets filled with mostly non-specific fibrovascular tissue^31^,^32^.

Here we have overcome the lack of adipogenic stimulus by injecting a small volume of lipoaspirate with no additional growth factors, cell transplantation or ligated vascular pedicles and introducing a completely novel prevascularisation technique that uses the patient’s own body as a bioreactor and a source of blood vessels. Based on our surgical team’s expertise and a literature search^23^,^24^,^25^,^26^, it was determined that 4 cm^3^ of adipose tissue is close to the maximum amount of fat that can be safely harvested from patients with a low body fat index. In terms of percentage, it represents 5.3% of total volume of the scaffold at the time of implantation.

The delayed lipo-injection technique allowed the formation of a bed of vascular and connective tissue within the scaffold volume. The presence of such a vascular and connective tissue has previously been found to support early adipogenesis, provided sufficient mesenchymal stem cells or adipose progenitor cells have been recruited to the implantation site^32^,^33^. In our study the adipose tissue, when injected into the already prevascularised scaffold, remained stably within the implantation sites with no sign of tissue necrosis. Over a period of 24 weeks, we estimated a fold increase in adipose tissue volume of 4.95 ± 0.31 in case of lipoaspirate-only and 6.1 ± 0.62 in case of prevascularisation + lipoaspirate group.

Scaffold degradation kinetics also plays a major role in the regeneration process. After implantation, continuous cell and tissue remodelling is essential to achieve and maintain stable biomechanical conditions, vascularization, and integration within the host site^34^. Importantly, biodegradable scaffolds are expected to stimulate and support both the onset and the continuance of tissue ingrowth as well as subsequent remodelling and maturation by providing suitable mechanical properties and external and internal geometrical shapes. Therefore, it is essential to understand and control their degradation process for successful tissue formation, remodelling and maturation at the defect site^35^. In the early days of tissue engineering, it was believed that scaffolds should degrade at the same rate as tissue growth^36^. This paradigm has been challenged in recent years it is now believed that tissue ingrowth and maturation differs temporally from tissue to tissue and tissue ingrowth does not equate to tissue maturation and remodelling^37^. Therefore, a defect filled with immature tissue should not be considered as “regenerated”. In keeping with this paradigm, our previously undertaken small animal study^38^ as well as the presented study has indicated that a scaffold made out slow-degrading polymer such as mPCL allows to maintain the mechanical properties for a sufficient period of time for large volume adipose tissue not only to show signs of regeneration but also to remodel at least twice and therefore present a mature tissue hemostasis at the time the scaffold shows any signs of mechanical degradation and/or mass loss.

The final breast tissue composition requirements also depend upon the surgical procedure. If the surgery is done mainly for aesthetic breast augmentation, one of the main goals is to maintain the natural tactile sensation of the breast. For this reason, the regenerated tissue needs to consist mainly of adipose tissue with smaller amounts of organised connective tissue in order to mimic the natural tissue morphology of the breast region. In case of post-mastectomy breast reconstruction, one of the major goals is to prevent a cancer relapse. In this case, adipose progenitor cells infiltrating into the scaffold may stimulate breast cancer recurrence via HGF/c-Met signalling^39^. Therefore, a majority of the regenerated tissue needs to be composed of highly organised connective tissue. The results of this study have indicated that the morphology of the regenerated tissue can be reproducibly controlled depending on initial scaffold treatment strategy (empty scaffold vs prevasculartion + lipoaspirate)–whereby empty scaffolds yield highly organised connective tissue whereas scaffolds containing lipoaspirate yield tissue rich in adipose tissue. In this way, scaffolds can truly be tailored for either an aesthetic augmentation procedure or a total reconstruction procedure.

We hypothesize that the mechanical properties of the constructs played a significant role in the neoadipogenesis process^40^. Contrary to musculoskeletal systems, where tissue such as bone and muscle grow in response to mechanical forces^40^,^41^, adipogenesis seems to be inhibited by mechanical forces^42^. The scaffolds used in this study had a stiffness value that was 3 orders of magnitude higher than native breast tissue^43^. We surmise that such the design and material properties would exert a shielding effect on the newly formed adipose tissue and reduced the effects of the compressive, tensile and shear forces acting on the fat tissue based on the body weight of the animal during rest in the laying position. This decreased mechanical stimuli would have allowed the cells to maintain a round morphology which, in turn, would have promoted adipogenesis of the adipose progenitor cells^44^.

From a clinical point of view, the stiffness of the scaffolds must also be made dependent upon their placement. In case of most cosmetic augmentations whereby the implants are placed in a subglandular pocket, the scaffold needs to remain elastomeric and flexible so as to not cause patient discomfort; whereas in case of most post-mastectomy breast reconstruction procedures whereby the implants are placed in a submuscular pocket and no other supporting tissue remains, stiffer implants are required in order to counteract the compression forces from the muscle^45^. Indeed, the role that mechanical forces play in the regeneration of high volumes of fat tissue warrants further research and we plan to study the design and additive biomanufacture of scaffolds with a range of mechanical properties in future experiments.

Non-specific localised low-grade granulomatose reactions were observed in the vicinity of the localised scaffold strands, consistent with previous reports^30^. A granuloma is an organised collection of macrophages^46^. While the roles of macrophages in angiogenesis are not yet completely understood, various research groups have shown that macrophages have the potential to contribute in angiogenesis^47^,^48^,^49^. More specifically, M1 macrophages secrete VEGF which initiates the process of angiogenesis, M2a macrophages secrete PDGF-BB known to be involved in later stages of angiogenesis, while M2c macrophages secrete high levels of MMP-9 known to have a role in remodelling of vasculature^50^. It has also been reported in the literature that macrophages can secrete alpha smooth muscle actin and can transdifferentiate into smooth muscle cells^51^,^52^. All groups showed accumulation of smooth muscle tissue around the scaffold strands (Fig. 8a–c) which can perhaps be taken as an indirect evidence of the fact that macrophages may have played a role in angiogenesis and consequently higher adipogenesis.

While no major outward signs of chronic inflammation were observed clinically or in the gross morphology of the constructs, lymphatic structures and leucocytes were detected in the histology of all groups–which is to be expected because the study used an immunocompetent animal model. mPCL is used in a wide range of FDA approved and CE-marked medical devices and implants and been proven in multiple independent studies to be an excellent biodegradable polymer for marinating mechanical properties over a long period of time and degradation and mass loss profile which does not cause an irreversible tissue reaction due to the production of a large amount of acidic by-products^15^,^53^,^54^,^55^,^56^. The increased leucocyte count may be explained by the fact that during the lipoaspiration process, adipose cells may have form non-viable aggregates in the syringe which, when injected into the scaffold, triggered an auto-immune reaction from the host aiming to break them down, ultimately leading to the ingression of lymphatic vessels.

Limitations of this study include the scaffold design. In this study, adipose tissue was injected directly into the vascular bed which may have caused localised tissue disruption. While such a tissue disruption may prove to enhance vascularisation^57^, this phenomenon cannot be controlled within a clinical setting. Therefore, future scaffold designs should contain specialised channels which can act as recipients for the lipoaspirate via minimal invasive surgery.

The presented study was based on a foundation of previous small animal studies and clinical observations^30^,^58^ and provided proof of principle that the prevascularisation and delayed fat injection techniques can be used for efficient regeneration of large volumes of adipose tissue for long periods of time. This work provides an important validation point for the regeneration of clinically relevant volumes of vascular-rich tissues in post-mastectomy breast reconstruction surgeries paving the way for future human clinical trials.

## Materials and Methods

### Study Design and sample size rationale

The presented study is a randomised and blinded animal study evaluating the adipose tissue regenerative potential of large 75 cm^3^ biodegradable scaffolds for 24-weeks using a subglandular swine animal model.

3 experimental groups were included in this study:

1. Empty scaffold (negative control).
2. Scaffold containing 4 cm^3^ lipoaspirate.
3. Empty scaffold + 2 week prevascularisation period. After 2 weeks of prevascularisation, 4 cm^3^ of lipoaspirate was injected into scaffolds.

The primary endpoint evaluated was the percentage of adipose tissue area compared to overall tissue area (AA/TA). We wished to detect no statistically significant difference in mean AA/TA between the experimental groups (prevascularisation + lipoaspirate and lipoaspirate-only groups) and the healthy breast tissue group (<10% difference in means). At the same time, we wished to detect a statistically significant difference between the AA/TA of negative control (empty scaffold) group and healthy breast tissue group. For an expected standard deviation of 5 (5 point scale), a sample size of 12 used in this study gives a statistical power of 85.7%. Statistical Power calculations performed using Researcher’s Toolkit Statistical Power Calculator (DSS Research, Fort Worth, USA).

#### Rules for stopping data collection

Data collection would be stopped and the scaffolds would be excluded from further analysis if one of the two following conditions were met (all signs verified by experienced plastic and veterinary surgeons):

1. Detection of infection.
2. Long-standing signs of haematoma or seroma.

#### Selection of endpoint

Since adipose tissue undergoes remodelling multiple times during the wound healing process, in this study a primary endpoint of 24 weeks was chosen to be adequate in terms of addressing tissue permanence mechanisms^59^,^60^,^61^,^62^.

#### Randomisation and Blinding

Two study parameters were randomised

1. Allocation of a scaffold to an experimental group.
2. Allocation of a scaffold to a subglandular pocket.

For both parameters, randomisation sequence was created using Excel 2010 (Microsoft, Redmond, USA) with a 1:1 allocation using random block sizes of 2 and 4 by an independent researcher. Except for the plastic surgeon operating on the animals, all researchers were kept blind to the allocation of scaffold and subglandular pockets to the experimental groups. Geographical separation ensured minimal contact between the operating surgeon and the researcher performing histological and qualitative analyses. Upon explantation, the operating surgeon coded each scaffold with an ID (JT-n; where n = 1 to 12) and kept the key hidden from the researchers performing downstream analyses. The key was revealed to the researchers only upon completion of the data analysis. In summary, all study outcomes were assessed in a blinded manner.

### Design & Fabrication of scaffolds

Additively manufactured hemisphere-shaped polycaprolactone-based scaffolds were designed and manufactured by Osteopore International Pte Ltd (Singapore). All scaffolds were produced using medical-grade polycaprolactone adhering to ISO 11137 (Sterilisation), 13485 (Quality Systems), 11607 (Packaging), and 14644-1 (Clean Room) standards.

### *In vivo* implantation into minipigs

The animal experiments were performed under GMP conditions at PWG Laboratories, Singapore with ethical approval from PWG Laboratories which, in turn, is maintained in accordance with NIH Guide for the Care and Use of Laboratory Animals. Two female adult immunocompetent minipigs were used in this study. The operation was performed under general anaesthesia, following the standard protocol of sterility requirements for breast augmentation procedures. Careful homeostasis was also maintained throughout the surgical procedure. 3 separate subglandular pockets were created on each side of the mammary region via a longitudinal incision. 6 implants were randomly placed in each animal.

In groups 2 and 3, a midline incision was made and adipose tissue was obtained via the Tulip system (Tulip Medical Products, San Diego, USA). Lipoaspirate was harvested from the abdominal fat region using the TULIP Technique. The freshly harvested Lipoaspirate was transferred into 10 ml syringes. The oil phase and the blood serous fluid were decanted following the currently performed clinical practices. Fat was then injected using a sterile and disposable 10-cm^3^ Tulip cell-friendly injector into the scaffold pores.

After the placement of the implants each pocket was closed with absorbable Vicryl sutures, such that the implants were fixed stably and had no contact to each other. Finally, the skin was sutured with interrupted 2.0 Ethilon sutures.

### Histological and Histomorphometrical analyses

#### Hematoxylin & Eosin (H & E)

Implants were harvested from the minipigs after 24 weeks and TECs were fixed with 4% PFA, cut into 10 mm × 10 mm cube sections, dehydrated and embedded in paraffin using a tissue processor (Excelsior ES, Thermo Scientific, Waltham, USA). Constructs were horizontally sliced to 5 μm, deparaffinised with Xylene, rehydrated with a decreasing series of ethanol and stained with H & E. Stained slides were scanned with a BIOREVO BZ-9000 microscope (Keyence, Itasca, USA).

#### Masson’s Trichrome

The slides were deparaffinised with Xylene, rehydrated with a decreasing series of ethanol and re-fixed in Bouin’s solution at room temperature overnight. After rinsing in tap water for 10 minutes, the slides were stained in Weigert’s iron hematoxylin for 10 minutes, rinsed in running warm tap water, stained in Biebrich scarlet-acid fuchsin solution for 10 minutes and transferred directly into aniline blue solution and stained for 10 minutes. The slides were rinsed briefly in distilled water and differentiated into 1% acetic acid solution for 5 minutes.

### Histomorphometry

Histomorphometrical analyses were undertaken the Osteomeasure histomorphometry analysis system (Osteometrics Inc., Decatur, GA, USA). All measurements were performed blinded on 8 randomly chosen sections from each scaffold from each group (4 from the superficial regions and 4 from the deep regions). To determine the average adipose tissue area, the total area of the adipose tissue was first calculated (A). Secondly, the total area occupied by the scaffold struts was measured (S). Finally, the combined area of the tissue section was measured (C). The ratio of adipose tissue area to total tissue area (R) was calculated using the following formula^30^:


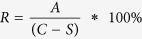


ImageJ (National Institutes of Health, MA, USA), in conjunction with Adipocyte Tools plugin developed by Montpellier RIO Imaging (Montpellier, France), was used for all automated calculations involving cell size distribution. The field of view (FOV) from each histological section was kept uniform. Background was first removed from each histological section by the pre-processing macro within the Adipocyte Tools plugin using the thresholding method. Minimum size of each cell was chosen to be 80 μm, maximum size as 800 μm and the number of dilates were set to be 10. These threshold values were kept constant across all samples and groups. The same threshold was also chosen to automatically set regions of interest (ROI) around the adipose cells. The automated method generated a small number of ROI artefacts. Artefacts that could be detected visually were manually removed. In order to remove the remaining artefacts, 10% of the smallest and 10% of the largest ROIs were excluded from any further analysis.

In order to calculate the blood vessel density, all blood vessels that showed red erythrocytes within the lumen were counted. The number of blood vessels was divided by the total tissue area to get the density. Values based on 4 stitched microphotographs from each scaffold per experimental condition.

#### Estimation of adipose volume in TEC

Since the entire volume of the scaffold was filled with host tissue, it is reasonable to conclude that each scaffold held 60 cm^3^ of total tissue volume at the end of the implantation period (75 cm^3^ total volume × 80% porosity = 60 cm^3^ volume available for tissue growth; scaffold degradation has not been taken into account as the mass loss starts only after 12–18 months^53^).

The relative adipose tissue fraction values shown in Fig. 8(g) have been calculated from 8 randomly chosen tissue sections, each 40 mm × 25 mm in dimensions. The estimated volume fraction of adipose tissue in each group was extrapolated from these adipose tissue area fraction values.

### Statistical analysis

All data are represented as mean ± SD and are subjected to one-way analyses of variance (one-way ANOVA) and Tukey’s post-hoc test (Prism 6, GraphPad, San Diego, USA). Significance levels were set at *p* < 0.05. All error bars represent standard deviation.

## Additional Information

**How to cite this article**: Chhaya, M. P. *et al.* Transformation of Breast Reconstruction via Additive Biomanufacturing. *Sci. Rep.* **6**, 28030; doi: 10.1038/srep28030 (2016).

## Supplementary Material

Supplementary Information

## Figures and Tables

**Figure 1:**
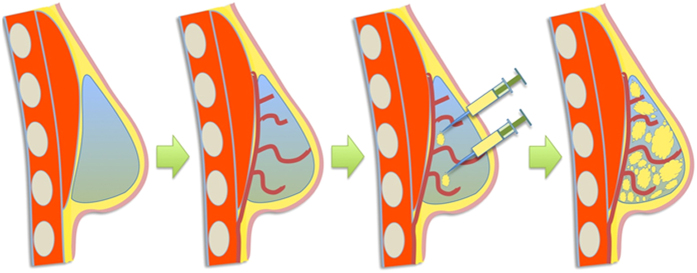
Overall concept of the prevascularisation and delayed fat injection concept. Empty scaffold is first implanted at the breast region without the addition of any cells or growth factors. Over the next 2–3 weeks, connective tissue and vasculature invades within the scaffold volume forming a bed of capillaries within the pores. Fat is then injected into the pores of the scaffold. Owing to the presence of the pre-formed vascular bed would allow the fat to remain stable at the implantation sites.
