# Supplementary material for: Transformation of Breast Reconstruction via Additive Biomanufacturing
Source: Sci Rep. 2016 Jun 15;6:28030. doi: 10.1038/srep28030 (PMC4908382; doi:10.1038/srep28030)
Supplement: Supplementary Information [file srep28030-s1.pdf]

Transformation of Breast Reconstruction via Additive Biomanufacturing.

Mohit P. Chhaya, Elizabeth R. Balmayor, Dietmar W. Hutmacher, Jan-Thorsten Schantz.

Supplementary Figure:

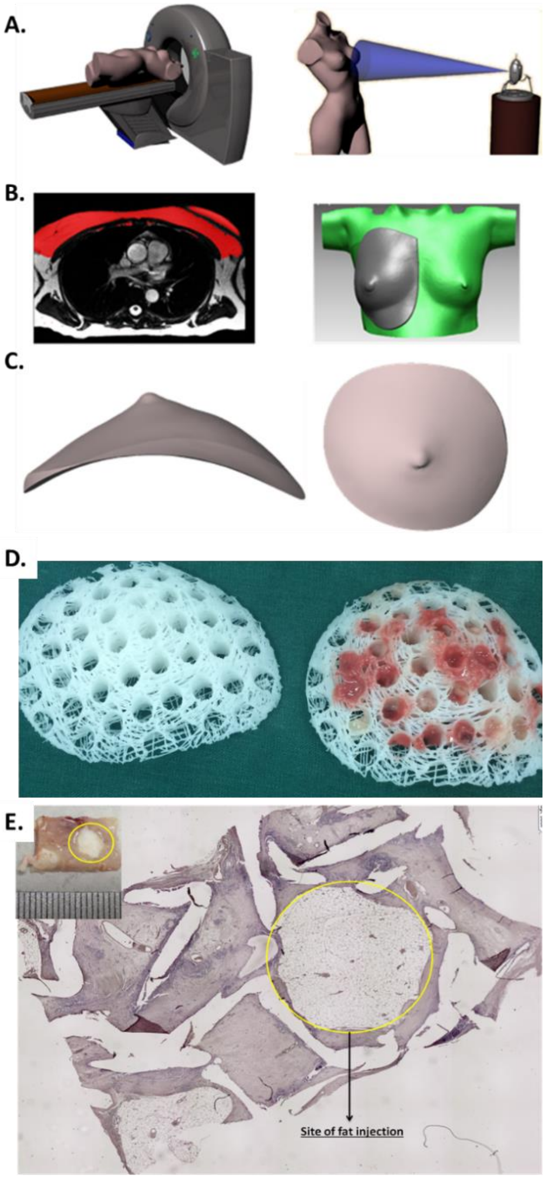

Supplementary Fig 1. Case study of a patient-specific breast scaffold fabricated using Additive Manufacturing techniques. The authors performed medical imaging (laser scanning and CT scan) on a patient with invasive ductal carcinoma (10) (A). A 3D model of the breast was generated from the imaging data set and was used to fabricate a 100cm3 patient-specific porous breast scaffold (B, C). The authors injected 40mL autologous fat into the scaffolds and implanted them into minipigs for a period of 6 months (D). (E) Hematoxylin and Eosin stained section of a scaffold explanted after 6 months implantation. Inset on top left shows a cut out of the area surrounding a randomly selected void filled with adipose tissue (adipose tissue encircled in yellow). The corresponding area in the histological section, also encircled in yellow, shows healthy well vascularised adipose tissue at the injection site with no signs of necrosis.
